# Fluid-Structure Interaction Simulations of Repaired Type A Aortic Dissection: a Comprehensive Comparison With Rigid Wall Models

**DOI:** 10.3389/fphys.2022.913457

**Published:** 2022-06-14

**Authors:** Yu Zhu, Saeed Mirsadraee, Ulrich Rosendahl, John Pepper, Xiao Yun Xu

**Affiliations:** ^1^ Department of Chemical Engineering, Imperial College London, London, United Kingdom; ^2^ National Heart and Lung Institute, Imperial College London, London, United Kingdom; ^3^ Department of Radiology, Royal Brompton and Harefield Hospitals, London, United Kingdom; ^4^ Department of Cardiac Surgery, Royal Brompton and Harefield Hospitals, London, United Kingdom

**Keywords:** fluid-structure interaction, repaired type A aortic dissection, hemodynamics, luminal pressure difference, wall shear stress

## Abstract

This study aimed to evaluate the effect of aortic wall compliance on intraluminal hemodynamics within surgically repaired type A aortic dissection (TAAD). Fully coupled two-way fluid-structure interaction (FSI) simulations were performed on two patient-specific post-surgery TAAD models reconstructed from computed tomography angiography images. Our FSI model incorporated prestress and different material properties for the aorta and graft. Computational results, including velocity, wall shear stress (WSS) and pressure difference between the true and false lumen, were compared between the FSI and rigid wall simulations. It was found that the FSI model predicted lower blood velocities and WSS along the dissected aorta. In particular, the area exposed to low time-averaged WSS ( ≤0.2 Pa ) was increased from 21 cm^2^ (rigid) to 38 cm^2^ (FSI) in patient 1 and from 35 cm^2^ (rigid) to 144 cm^2^ (FSI) in patient 2. FSI models also produced more disturbed flow where much larger regions presented with higher turbulence intensity as compared to the rigid wall models. The effect of wall compliance on pressure difference between the true and false lumen was insignificant, with the maximum difference between FSI and rigid models being less than 0.25 mmHg for the two patient-specific models. Comparisons of simulation results for models with different Young’s moduli revealed that a more compliant wall resulted in further reduction in velocity and WSS magnitudes because of increased displacements. This study demonstrated the importance of FSI simulation for accurate prediction of low WSS regions in surgically repaired TAAD, but a rigid wall computational fluid dynamics simulation would be sufficient for prediction of luminal pressure difference.

## Introduction

Aortic dissection (AD) occurs when the inner layer of the aortic wall tears and blood flows in between the inner and outer layers of the wall, developing a false lumen (FL) in the aortic wall alongside the original true lumen (TL). Based on the most widely used Standford classification system, AD can be divided into type A and B, depending on the location of primary entry tear: type A if the entry tear is located in the ascending aorta and the arch, whilst type B when the entry tear is situated in the descending aorta. Type A aortic dissection (TAAD) represents a more lethal condition than type B aortic dissection (TBAD), which requires urgent surgical intervention to reduce its life-threatening complications. An established surgical technique for the treatment of TAAD is to replace the ascending aorta that involves the primary entry tear with a synthetic graft. In spite of having the lowest reported perioperative risk and overall mortality (Westaby et al., 2002), this conservative surgical approach usually results in incomplete resection of re-entry tears in the arch and descending aorta, which increases the risk of late complications such as aneurysmal dilatation of the remaining dissected aorta (Halstead et al., 2007). With a persistent patent FL, 29.3% of patients were reported to die from rupture of the residual dissected aorta (DeBakey et al., 1982).

To prevent sudden aortic rupture and late death, efforts have been made to identify risk factors for aortic dilatation following TAAD repair. In addition to anatomical features, such as maximum aortic diameter and FL patency (Fattori et al., 2000; Halstead et al., 2007; Zierer et al., 2007; Rylski et al., 2017), certain hemodynamic parameters have been reported to correlate with progressive aortic dilatation, including luminal pressure difference between TL and FL (Zhu et al., 2021; Zhu et al., 2022) and flow velocities through tears (Shad et al., 2021). However, these hemodynamic parameters were obtained from computational fluid dynamics (CFD) simulations with a rigid wall assumption. In reality, the aorta expands and contracts in response to the pulsation of blood pressure, and recent computational studies of TBAD have shown the influence of wall compliance on predicted luminal pressure and volumetric flow rate (Bonfanti et al., 2017; Chong et al., 2020). Therefore, it is necessary to evaluate the dynamic effects of moving wall on blood flow in the surgically repaired TAAD by fluid-structure interaction (FSI) simulation.

Building an FSI model of AD is very challenging since the wall thickness and material properties vary in different components of the vessel wall and are difficult to measure *in vivo*. Additionally, the complexity of the model will demand extensive computational resources for FSI simulation. In the last decade, only a few FSI simulations of TBAD have been reported, either by using simplified and idealized models (Chen et al., 2016; Ryzhakov et al., 2019; Chong et al., 2020) or patient-specific geometries (Alimohammadi et al., 2015; Qiao et al., 2015; Qiao et al., 2019; Zimmermann et al., 2021). Chong et al. (2020) assessed the effect of intimal flap motion on flow in TBAD. Despite using an idealized model, the maximum flap motion reached 4.6 mm and thus significantly altered the predicted hemodynamic parameters as compared to the rigid wall models. In two patient-specific TBAD FSI studies, the obtained results were also compared with those from rigid wall models (Alimohammadi et al., 2015; Qiao et al., 2019). These studies demonstrated that although spatial distributions of time-averaged wall shear stress (TAWSS) obtained with FSI and rigid wall models had similar trend, there were marked differences in the predicted oscillatory shear index.

The aforementioned FSI studies focused on TBAD. Only two studies were found that involved FSI simulations of residual TBAD patients with the ascending aortas being replaced with synthetic grafts (Bäumler et al., 2020; Khannous et al., 2020). Bäumler et al. (2020) developed a sophisticated FSI model by including pre-stress, external tissue support, geometry tethering, as well as a regionally defined flap elasticity with variable values. Their results showed an overall good agreement with 4-D magnetic resonance imaging (MRI) data. However, the synthetic graft, which is much stiffer than the aortic wall, was not modelled in their study. Moreover, comparison with the corresponding rigid wall CFD simulation results was not reported, which is in fact of great interest.

In this study, to gain more knowledge of how wall compliance may influence intraluminal hemodynamics, FSI simulations have been performed on two repaired TAAD models. Our FSI model not only incorporates pre-stress but also applies different material properties and wall thickness for the aorta and graft. Additional simulations have been carried out to evaluate the effects of dissection wall stiffness. The obtained results are compared with those from the rigid wall models of the same patients, with particular attention to pressure difference between the TL and FL.

## Materials and Methods

### Patients’ Information

Two patients with repaired TAAD were retrospectively selected from the validated database of patients at the Royal Brompton and Harefield hospitals, United Kingdom. The first patient was a 44-year-old male who underwent graft replacement of the ascending aorta. Computed tomography angiography (CTA) in this patient was performed on a Sensation 64 scanner (Siemens Medical Solutions, Germany), where the slice thickness and increment of CTA images were 1-mm. The second patient was a 51-year-old male, who underwent replacement of the aortic valve and ascending aorta for TAAD 4 years prior to CTA scan. This patient was examined by a SOMATOM Definition Flash scanner (Siemens Medical Solutions, Germany), and the images were reconstructed with 0.75-mm slice thickness and 0.5-mm slice increment. All medical data included in this study complied with the Declaration of Helsinki and were approved by the Institutional committee of Health Research Authority (HRA) and Health and Care Research Wales (HCRW). Need for patients’ informed consent was waived.

### Geometry Reconstruction and Mesh Generation

**FIGURE 1:**
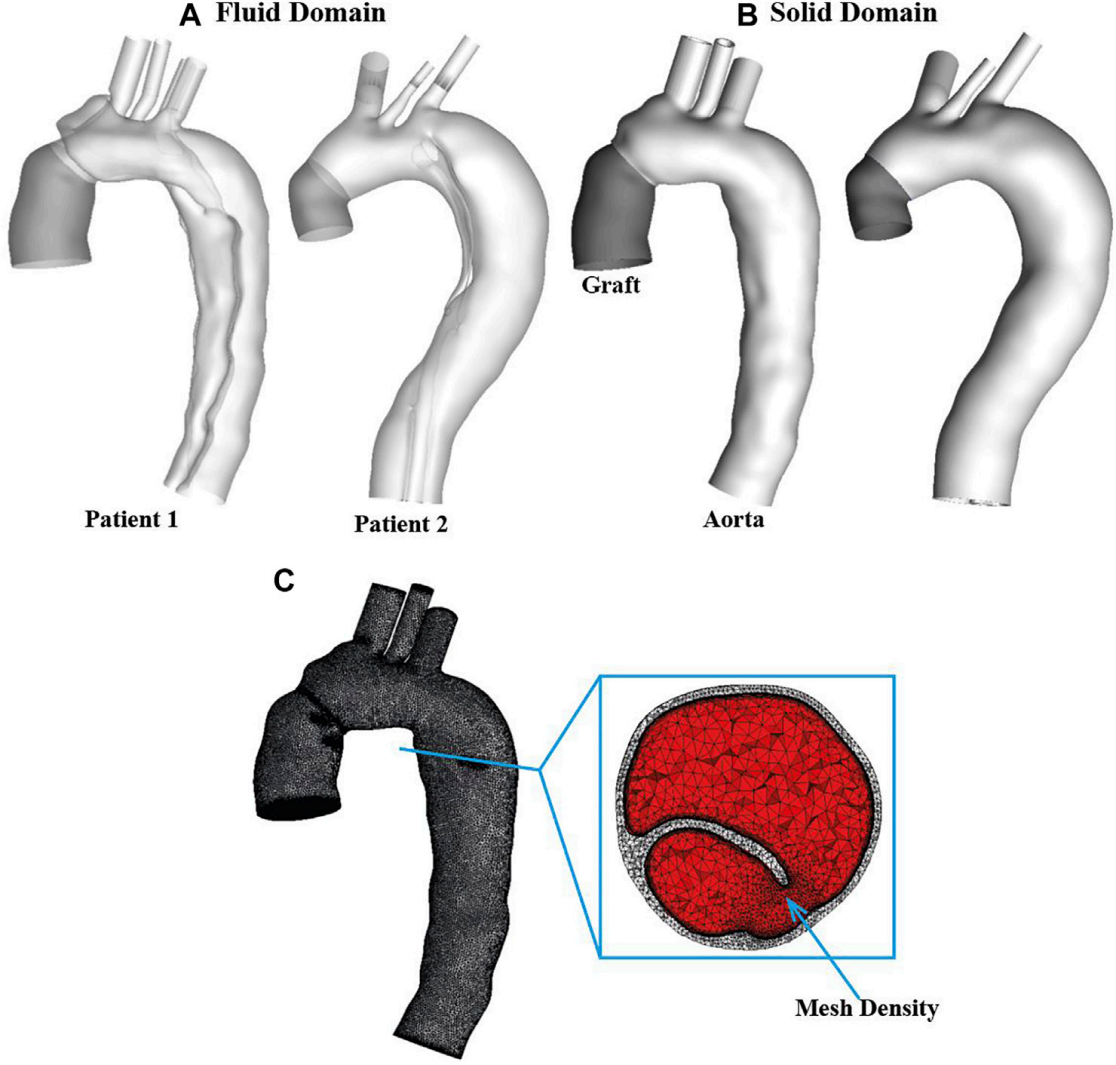
Two patient-specific type A aortic dissection models for reconstructed for **(A)** fluid domain and **(B)** structural domain. Graft is shown in dark grey while the aorta is shown in light grey. Detailed mesh elements are shown for **(C)** fluid-structure interaction model. It can be seen clearly that the solid domain (light grey) was discretized into tetrahedral elements, while the fluid domain (red) was meshed with a tetrahedral core and prismatic layers at the wall. Moreover, a region with mesh density is indicated by arrow.

The patient-specific geometries of post-surgical TAAD were reconstructed from the CTA images using Mimics 20.0 (Materialise, Leuven, Belgium). For each patient, the computational model was created from the aortic sinotubular junction to the level of diaphragm. Three main arch branches were also included in the reconstructions, as shown in Figure 1A. The reconstructed geometry was not only used as the 3-D fluid domain but also provided the inner surface of the wall in the FSI model. The wall structural domain was created by uniformly extruding the undissected equivalent of the inner wall by 1.4 mm for the aorta and the intimal flap (van Puyvelde et al., 2016) and by 0.65 mm for the Dacron graft (Nagano et al., 2007), as shown in Figure 1B.

The fluid and solid domains were then meshed separately using ANSYS ICEM CFD 19.2 (ANSYS, Canonsburg, PA, United States). As shown in Figure 1C, for each geometry, the structural domain was discretized into unstructured tetrahedral meshes comprising approximately 0.85 and 0.1 million elements for the aorta and graft, respectively, whereas the fluid domain was meshed with a tetrahedral core and 10 prismatic layers at the wall comprising of around 1.5 million elements. Mesh sensitivity tests were carried out for both fluid and solid domains and the corresponding results are summarized in Supplementary Material S1A.

### Fluid Domain

At the inlet of fluid domain, a scaled patient-specific flow waveform was imposed along with the assumption of a flat velocity profile. A 3-element windkessel model (3-EWM) was imposed at each outlet. Details of the applied boundary conditions for flow analysis can be found in our previous study (Zhu et al., 2021). Blood was assumed to be incompressible and Newtonian with a constant density of 1,060 kg/m^3^ and dynamic viscosity of 0.004 Pa.s. Flow in a dissected aorta is likely to become transitional or turbulent induced by geometric features, such as a narrow tear or highly compressed TL. To account for possible turbulence behavior, the hybrid k−∈/k−ω shear stress transport transitional (SST-Tran) model (Menter et al., 2006) was applied in this study, with a turbulence intensity of 1% being specified at the model inlet, similar to previous studies (Cheng et al., 2010; Chen et al., 2013). A fixed time-step of 0.005 s was chosen based on the time-step sensitivity tests (Supplementary Material S1B). For the purpose of comparison, CFD simulations with a rigid wall assumption were carried out with the same fluid mesh and simulation settings. All simulations were run over 4 cardiac cycles for patient 1 and 9 cardiac cycles for patient 2 to achieve a periodic solution, and results obtained in the last cycle were used for detailed analysis.

### Structural Domain

Both the graft and aortic walls were modelled as isotropic, homogenous and linear elastic materials. The Dacron graft used to replace the ascending aorta is made of polyethylene terephthalate (PET), with a reported Poisson’s ratio of 0.3 and Young’s modulus of 7.8 MPa (Weltert et al., 2009). A Young’s modulus of 1.3 MPa was found to be comparable to *in vivo* aortic wall compliance (Zimmermann et al., 2021) and thus adopted for the dissected aortic wall and intimal flap in this study. To assess the impact of aortic stiffness on the predicted results, additional FSI simulations were run for patient 1 with different Young’s moduli: 1.08 and 2 MPa, representing more compliant (Patient 1A) and stiffer (Patient 1B) aortic wall behaviors, respectively. Both values were used in previous FSI studies of aorta (Wang and Li, 2011; Chen et al., 2016; Ryzhakov et al., 2019; Qiao et al., 2021). Moreover, a Poisson’s ratio of 0.49 was applied to the aortic wall, even though it is usually considered incompressible (Bäumler et al., 2020). The aortic root motion was neglected in this study. Therefore, zero-displacement constraints were applied at the inlet, at the distal ends of three arch vessels, as well as at the mid-descending aorta of the structural domain. Rayleigh damping (α=50, β=0.1) was also applied to account for support provided by the surrounding tissue.

The CTA images of both patients were obtained at diastole and thus the reconstructed geometries represented the aorta configurations under a diastolic intraluminal blood pressure, necessitating the estimation of prestress to account for physiological initial loading state. The prestress was calculated using Ansys Static Structural solver (ANSYS, Canonsburg, PA, United States), based on the method described by Votta et al. (2017) and modified by Kan et al. (2021). Briefly, pressure distributions from the last time-step of the rigid wall CFD simulation were exported and then mapped onto the internal surface of the aortic wall model. The structural domain was deformed, and the corresponding Cauchy stress tensor was exported, which was then prescribed as initial stress state for the next simulation. This procedure was repeated until the maximum deformation of the structural domain was less than 0.5 mm under a diastolic pressure loading. Therefore, the prestress tensor equivalent to the diastolic phase was obtained and applied in the FSI simulation. It should be mentioned that the same material property and setups as the final FSI simulation were used for prestress calculation.

### Fluid-Structure Coupling

The wall models of both patients were solved using ANSYS Transient Structure solver, while the pulsatile blood flow was solved using ANSYS CFX 19.2. The two-way FSI simulation was then performed using ANSYS system coupling (ANSYS, Canonsburg, PA, United States), which couples ANSYS Structure and ANSYS CFX through a partitioned approach.

The arbitrary Lagrangian-Eulerian (ALE) method was applied for FSI, which utilizes the best features of both, Lagrangian and Eulerian approaches, and combines them into one. The Lagrangian approach is typically adopted in solid mechanics to define the structural domain as each node of the computational element follows the associated material particle during motion. The Eulerian method is widely used in fluid dynamics as the computational element in the fluid domain is fixed in space and the continuum moves with respect to the grid. A FSI model can be considered as a combination of three coupled sub-problems: a geometry problem that defines a new reference configuration, namely, the ALE map; and fluid and solid problems which comprise the conservation equations for the fluid and solid, respectively (Crosetto et al., 2011).

In the ALE configuration, the continuity and momentum equations governing the blood flow are given as:

∇ ⋅v=0 (1)

$$\rho_{f}\frac{\partial v}{\partial t}+\;\rho_{f}\left[\left(v-\;d_{f}\right)\cdot \nabla v\right]=\;-\nabla p+\;\nabla \cdot \tau_{f}+F_{f}$$ (2)

where $\tau_{f}\;$ is the viscous stress tensor and $\nabla \cdot \tau_{f}=\;\mu \nabla^{2}v$ for Newtonian fluid flow where μ is the blood viscosity, ∇ is the divergence operator, v is the fluid velocity vector, $\rho_{f}$ is the fluid density, $F_{f}$ is the body force (per unit volume) acting on the fluid, *p* is the pressure and $d_{f}$ is the moving boundary velocity vector. The term $\left(v-\;d_{f}\right)$ refers to the relative velocity of the fluid with respect to the moving coordinate velocity.

The governing equation for the structural domain is given by the following momentum conservation equation:

$$\nabla \cdot \sigma_{s}+\;F_{s}=\;\rho_{s}{\ddot{d}}_{s}\;$$ (3)

where $\sigma_{s}$ is the solid stress tensor, $F_{s}$ is the force (per unit volume) acting on the solid, $\rho_{s}$ is the solid density, and ${\ddot{d}}_{s}$ is the local acceleration of the solid. The fluid and structural domains are then coupled at the FSI interface, where the following conditions are applied: 1) displacements of the fluid and structural domain must be compatible, 2) tractions at these boundaries must be at equilibrium, and 3) the no-slip condition is still valid.

$$u_{s}=\;u_{f}$$ (4)

$$\sigma_{s}{\hat{n}}_{s}=\;\sigma_{f}{\hat{n}}_{f}\;$$ (5)

$$\frac{\partial u_{f}}{\partial t}=v$$ (6)

where u is displacement vectors, with the subscript ***s*** indicating a property of solid and ***f*** of fluid, and $\sigma_{f}$ is the fluid stress tensor. Vector $\hat{n}$ is the boundary normal direction, and ${\hat{n}}_{s}=-{\hat{n}}_{f}$ at the fluid-solid interface.

To maintain the quality of the fluid mesh, the mesh was smoothed using displacement diffusion method with mesh stiffness being blended with distance and small volumes. A coupled time-step of 0.005 s was specified. Within each coupled time-step, the iterations were repeated until a maximum number of iterations was reached or until the data transferred between solvers and all field equations were converged. In the fluid model, the maximum root mean square (RMS) residual of 10^−5^ was specified, whereas in the structural model, the maximum RMS residual was set as 10^−3^. The simulation results were and analysed using CEI Ensight 10 (CEI Inc., Apex, NC, United States).

## Results

### Wall Displacement

**FIGURE 2:**
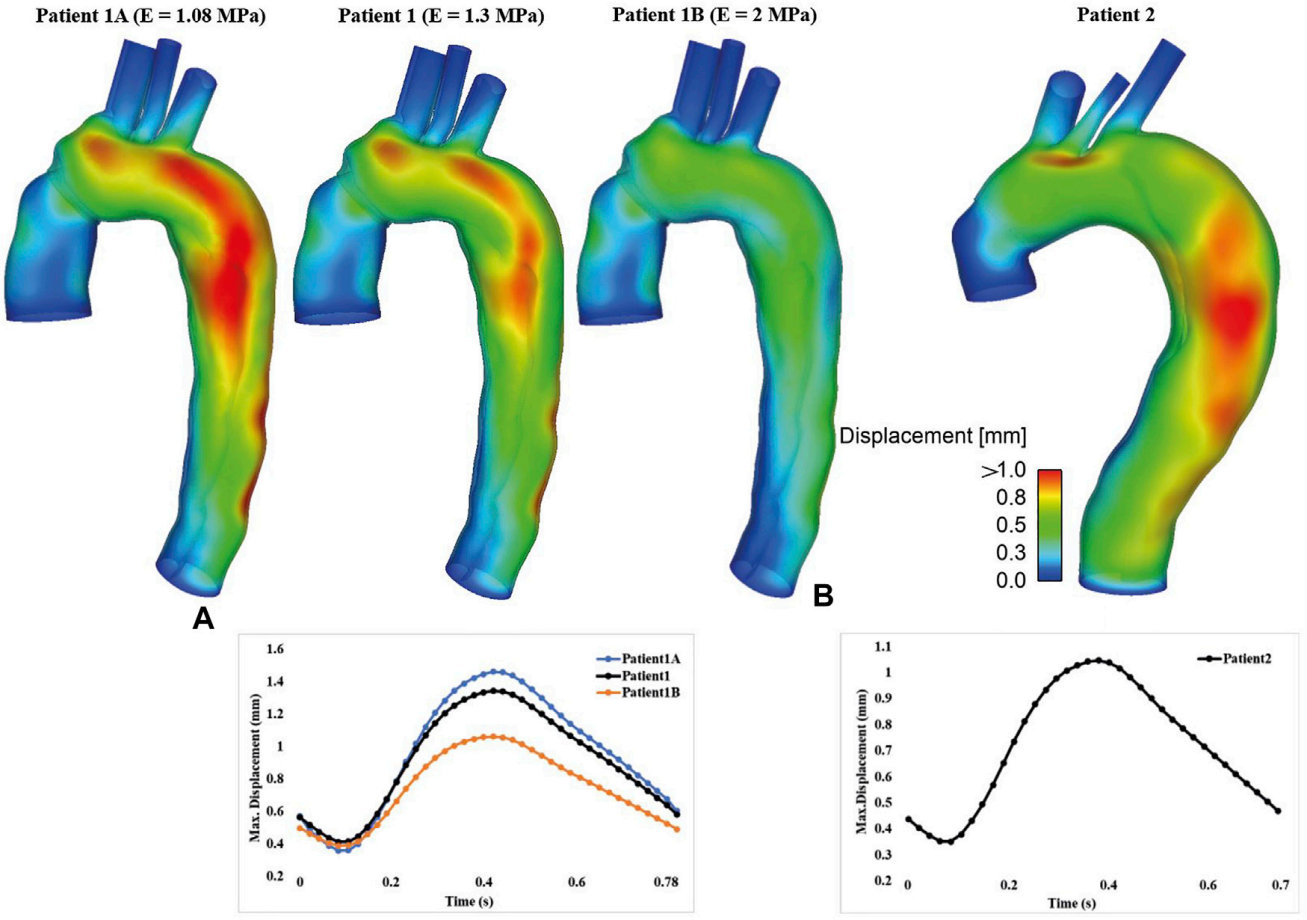
Spatial distribution of displacement (Top) and the corresponding temporal variations of maximum displacement (Bottom) are shown for **(A)** patient 1 and **(B)** patient 2. Moreover, displacement results for FSI models with different Young’s moduli were compared for patient 1.

The displacement contours of the aortic wall are shown in Figure 2 for all simulated FSI models at the time point when maximum displacement occurred, together with the corresponding maximum displacement waveforms over a cardiac cycle. In all cases, the aorta segment replaced by the synthetic graft and supra-aortic branches show very limited deformation, whereas noticeable displacements could be observed throughout the aortic arch and descending aorta. The maximum wall displacement occurred in the proximal descending aorta of both patients, with the values being 1.34 mm for patient 1 and 1.05 mm for patient 2. Multiple re-entry tears might result in less deformation in patient 2. Increasing aortic wall stiffness significantly reduced displacements over the entire cardiac cycle. The maximum displacement decreased from 1.46 to 0.97 mm when the Young’s modulus was doubled.

### Flow Patterns and Velocity Magnitudes

**FIGURE 3:**
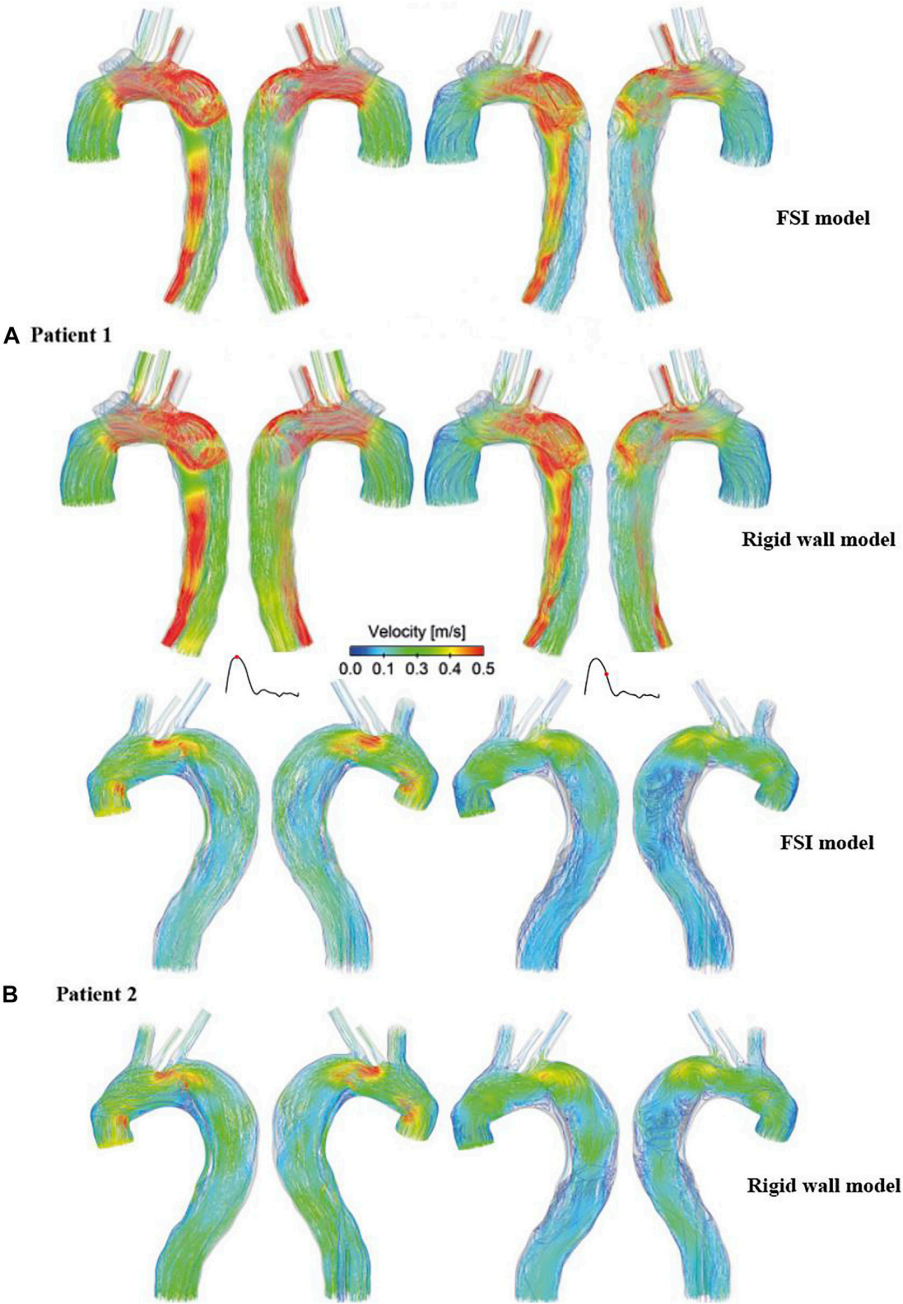
Comparison of instantaneous velocity streamlines obtained from the rigid wall models and FSI models of **(A)** patient 1 and **(B)** patient 2, at peak systole (left) and mid-systolic deceleration (right).

Figure 3 shows instantaneous velocity streamlines obtained with the FSI simulations and the corresponding rigid wall models. In general, flow patterns at the systolic and maximum flow deceleration time points were similar, with high velocities through the tears and in regions with narrowed lumen, such as the distal descending TL of patient 1. Flow patterns obtained from the FSI and rigid wall models were qualitatively the same and quantitatively comparable, where the FSI models produced slightly lower blood velocities in the distal thoracic aorta compared to the rigid wall models.

**FIGURE 4:**
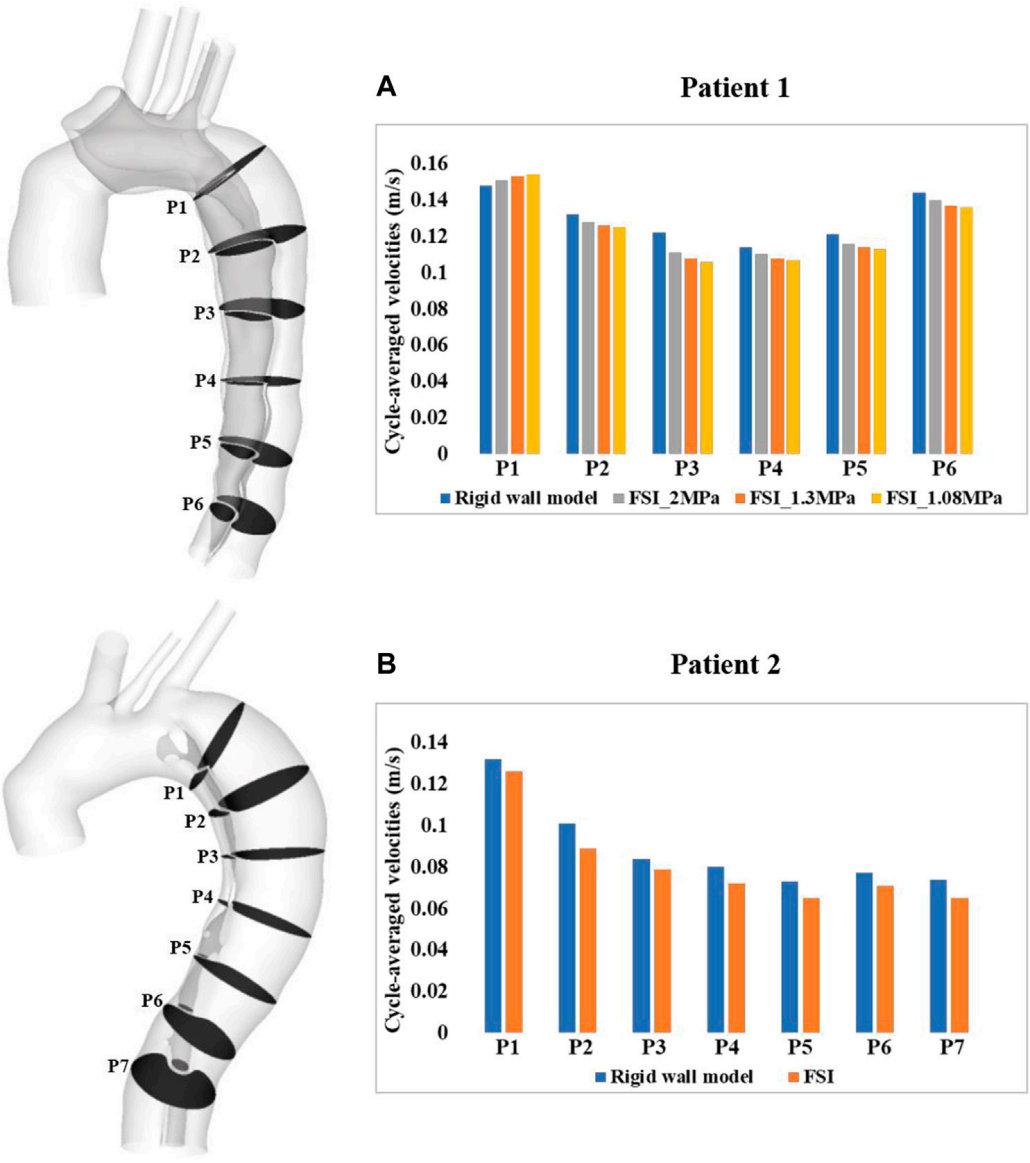
Quantitative comparison of cycle-averaged velocities at the selected cross-sectional planes along the aorta of **(A)** patient 1 and **(B)** patient 2. P1-P6/7 refer to cross-sectional planes along the centerlines of the dissected aorta. P1 is 2 cm distal from the origin of LSCA and P2-P6/7 are evenly spaced below P1 with an interval of 3 cm. Velocity magnitudes were also compared between the FSI models with different Young’s moduli in patient 1.

A more detailed quantitative comparison of velocity magnitudes was made between the rigid and FSI models. As shown in Figure 4, multiple cross-sectional planes were selected along the centerlines of the dissected aorta (six for patient 1 and seven for patient 2 depending on the aortic length), in order to calculate spatial-mean velocities, which were then averaged over a cardiac cycle and compared between different models. Quantitative comparisons revealed that FSI models generally predicted lower velocity magnitudes. This is expected since the aortic wall expands during systole, leading to an increase in lumen volume hence a reduction in velocities. Comparing FSI models of patient 1 with different Young’s moduli, a more compliant model produced lower velocity magnitudes, resulting from larger wall deformations, as shown in Figure 2.

### Flow Exchange Between True and False Lumen

**FIGURE 5:**
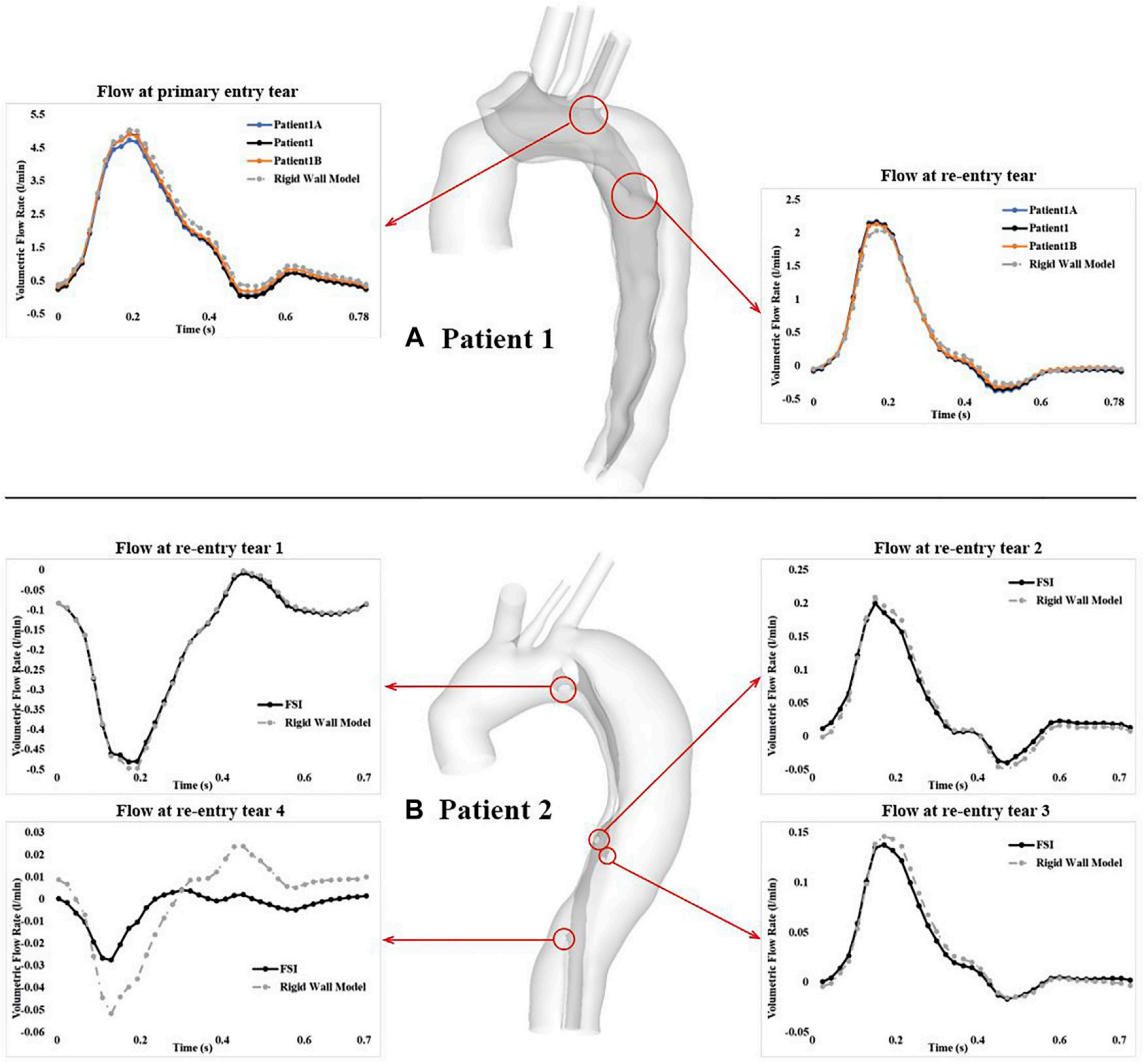
Comparison of volumetric flow rate over a cardiac cycle at the primary entry tear and re-entry tear of **(A)** patient 1, and at all 4 re-entry tears of **(B)** patient 2. Locations of all the tears are highlighted by the red cycles. Tear flows were also compared between the FSI models with different Young’s moduli in patient 1.

Figure 5 shows the comparison of volumetric flow rate at the primary entry tear and re-entry tear of patient 1, and at all re-entry tears of patient 2. Although FSI and rigid wall models displayed qualitatively similar trends in both patients, quantitative differences were observed. Over a cardiac cycle, the percentage of inflow passing through the primary entry tear and re-entry tear was 25.4% and 5.6%, respectively, in the FSI model of patient 1, and 28.5% and 6.1% for the rigid wall model. It was not possible to calculate the volumetric flow rate at the primary entry tear of patient 2 owing to its irregular shape. Nevertheless, −2.6%, 0.63%, 0.44%, and −0.06% of inflow passed through re-entry tears 1 to 4, respectively, in the FSI model, with the corresponding values being −2.6%, 0.6%, 0.83%, and −0.02% in the rigid wall model. It should also be noted that a positive value represents flow from the TL to FL, whereas a negative value indicates flow from the FL to TL. Moreover, increasing the aortic wall Young’s modulus from 1.08 to 2 MPa resulted in 6.9% and 5.1% increase in mean flow rate at the primary entry tear and re-entry tear, respectively.

### Wall Shear Stress

**FIGURE 6:**
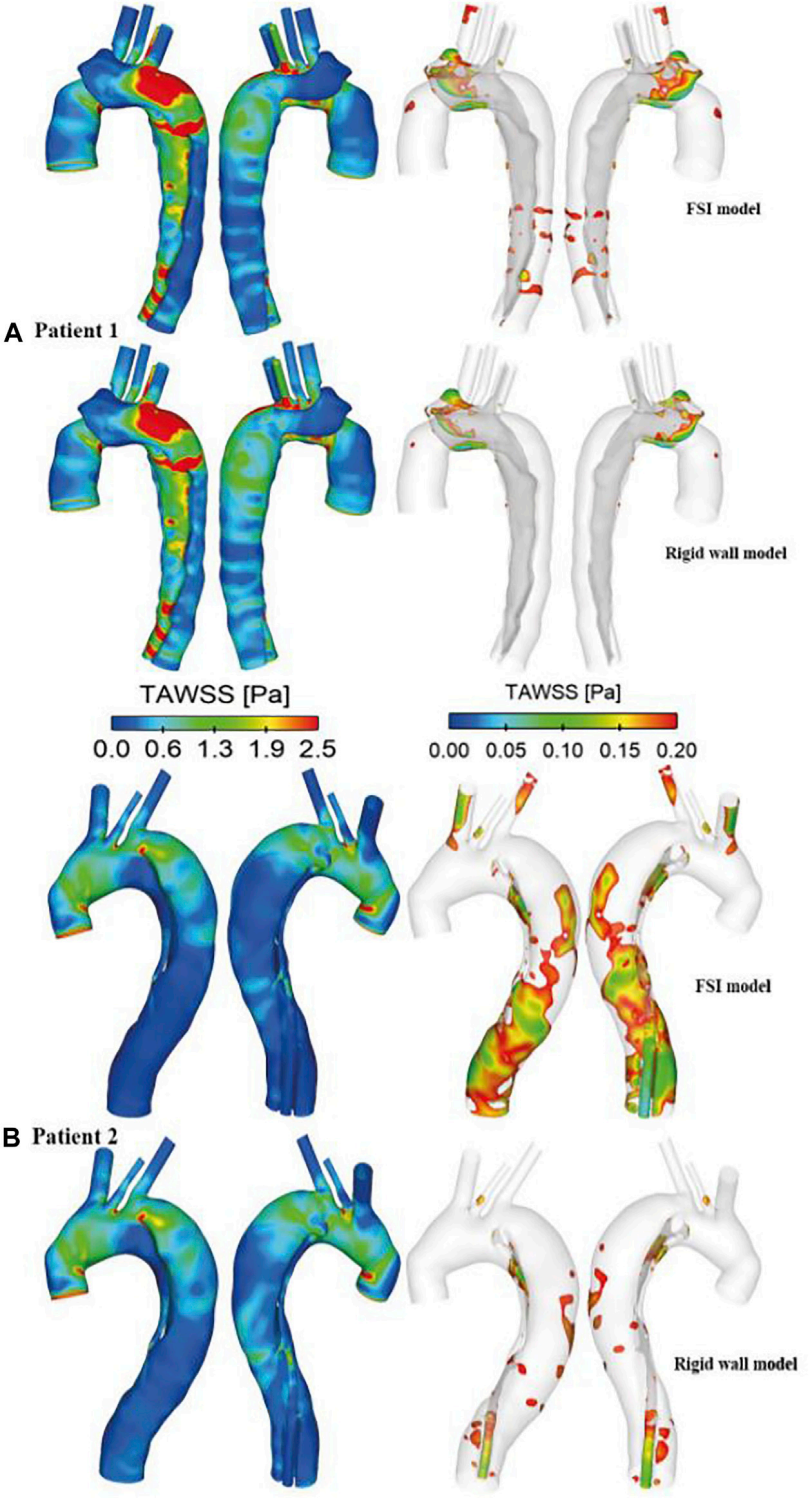
Comparison of time-averaged wall shear stress (TAWSS) distributions between the rigid wall and FSI models of **(A)** patient 1 and **(B)** patient 2. TAWSS distributions are displayed in different views to identify regions with high (>2.5 Pa, left) and low (<0.2 Pa, right) values.

TAWSS was calculated using Eq. 7.

$$TAWSS=\;\frac{1}{T}\;\overset{T}{\underset{0}{\int }}\left|\tau_{\omega }\right|dt$$ (7)

where T is the period of the cardiac cycle and $\tau_{\omega }$ is the instantaneous wall shear stress (WSS). Spatial distributions of TAWSS were compared between the FSI and rigid models, and the results are shown in Figure 6. Similar to flow patterns, the FSI and rigid wall models produced qualitatively similar results, with high WSS concentrated in the regions surrounding the tears. The maximum TAWSS were found on the edge of the primary tears in both cases. The peak TAWSS values are 20.5 and 21.3 Pa in the rigid wall model and FSI model of patient 1, respectively. On the contrary, the FSI model of patient 2 predicted slightly lower peak TAWSS value of 10.7 Pa, as compared to 11.1 Pa in the rigid model. Moreover, the regions with very low TAWSS values ( ≤0.2 Pa ) were also compared. The results revealed that FSI models predicted larger areas with low TAWSS in both patients, more obviously for the distal descending FL of patient 2 (patient 1: 21 cm^2^ rigid vs. 38 cm^2^ FSI; patient 2: 35 cm^2^ rigid vs. 144 cm^2^ FSI).

**FIGURE 7:**
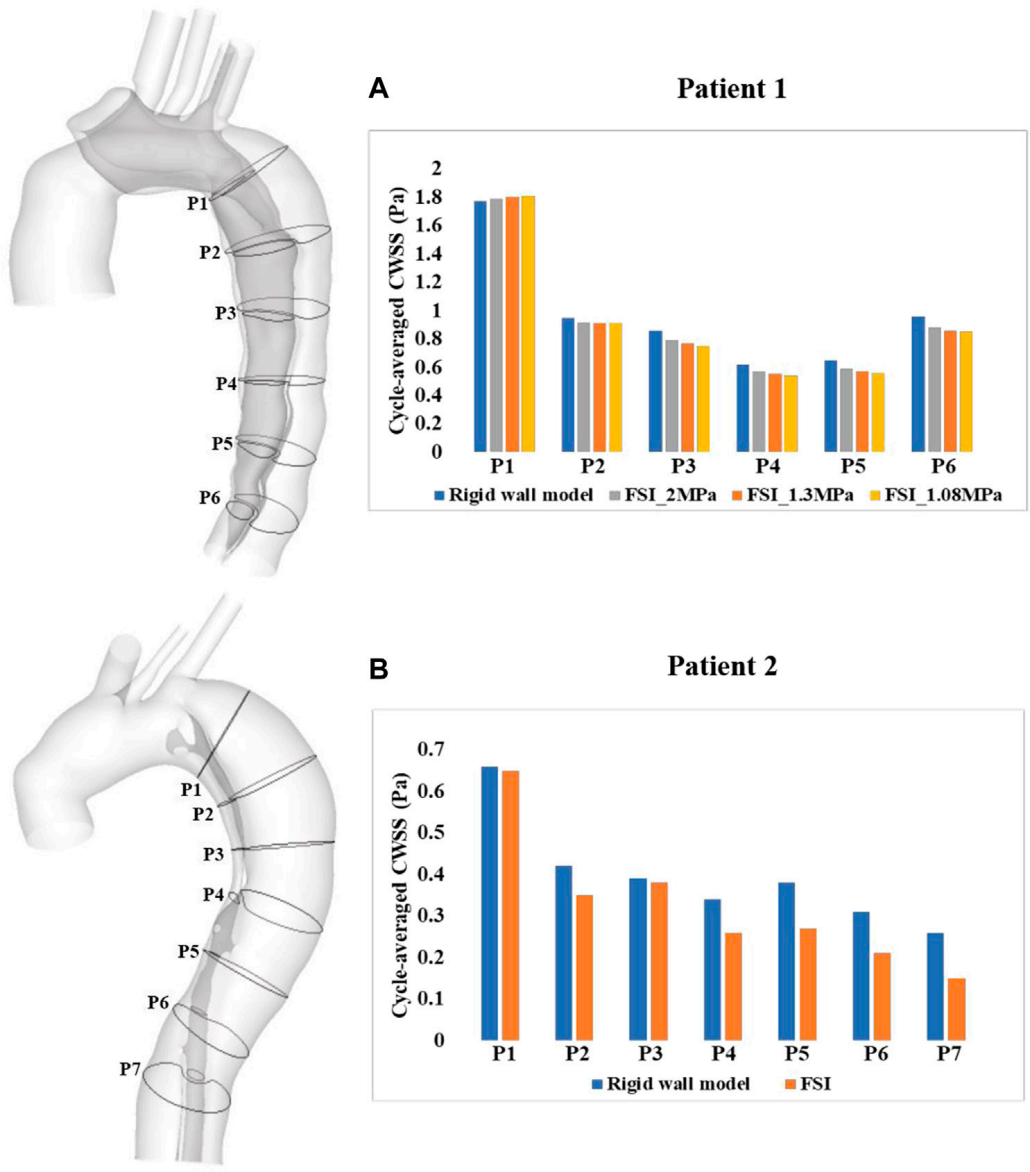
Quantitative comparison of cycle-averaged circumferential wall shear stress (CWSS), at the selected clips along the aorta of **(A)** patient 1 and **(B)** patient 2. P1-P6/7 refer to clips (intersection lines) between cross-sectional planes and the aortic walls. CWSS magnitudes were also compared between the FSI models with different Young’s moduli in patient 1.

A more detailed quantitative comparison of circumferential-averaged WSS (CWSS) was made between the rigid and FSI models, and the results are illustrated in Figure 7. The CWSS was evaluated as the spatial-averaged WSS along the intersection lines between the cross-sectional planes and the aortic walls including the flap, after which the cycle-averaged CWSS magnitudes were evaluated and compared for all the simulated models. Again, similar to velocity magnitudes, the FSI models of both patients produced lower CWSS values as a result of aorta expansion. Moreover, stiffer aortic wall led to slightly higher CWSS values, while the maximum difference among all cross-sectional clips between patient 1A (E = 1.08 MPa) and 1B (E = 2 MPa) is only 5.6%.

### Maximum Pressure Difference Between True and False Lumen

**FIGURE 8:**
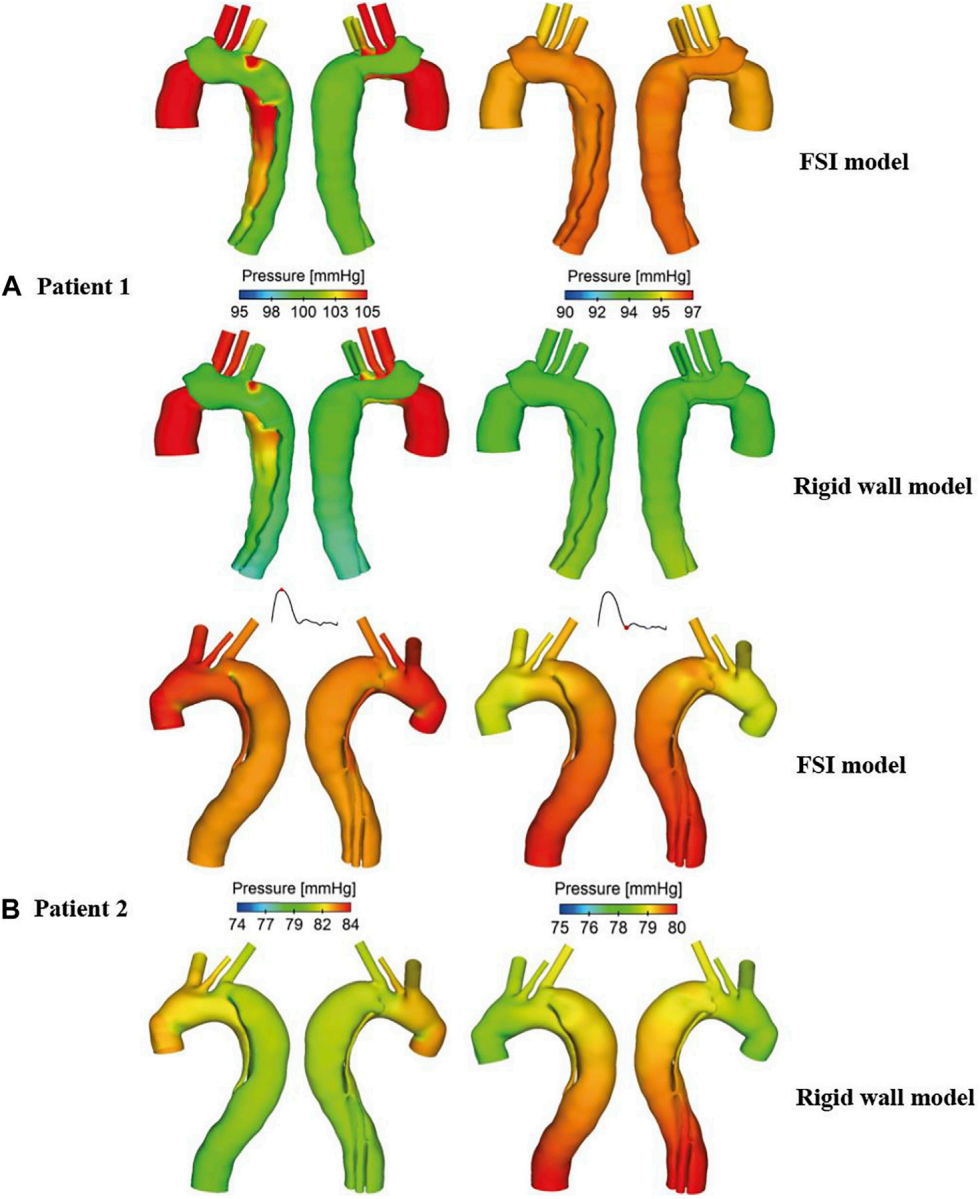
Comparison of pressure contours obtained from the rigid wall and FSI models of **(A)** patient 1 and **(B)** patient 2, at peak systole (left) and end systole (right).

Pressure distributions at two time points are shown in Figure 8. At peak systole, higher TL pressure can be observed throughout the descending aorta, especially in the proximal segment, while almost equal TL and FL pressures were found at end systole. Again, pressure distributions predicted by the FSI and rigid wall models were qualitatively comparable but quantitively different, where FSI models predicted higher wall pressures than the rigid wall models.

**FIGURE 9:**
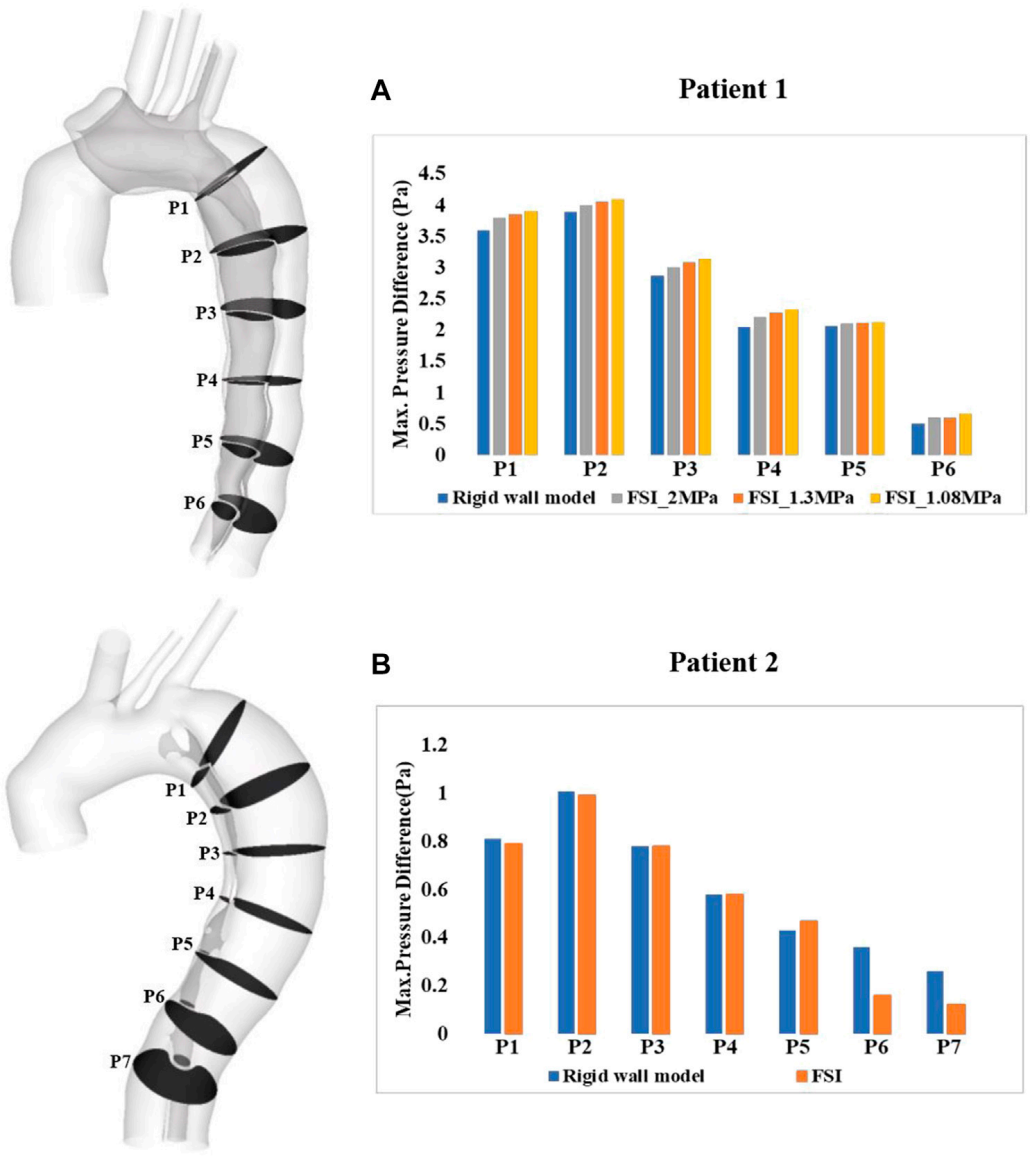
Quantitative comparison of pressure difference between the true and false lumen (P_TL_–P_FL_) at the selected cross-sectional planes along the aorta of **(A)** patient 1 and **(B)** patient 2. P_TL_–P_FL_ values were also compared between the FSI models with different Young’s modulus in patient 1.

Spatial mean pressures over a cardiac cycle were evaluated separately for the TL and FL at each cross-sectional plane. Then, differences between TL and FL pressures (PD_TL-FL_ = P_TL_–P_FL_) were calculated, and within each cross-sectional plane, the maximum PD_TL-FL_ over a cardiac cycle was determined. Figure 9 shows the quantitative comparison of the maximum luminal pressure differences predicted by different models. Irrespective of the location, incorporating wall compliance resulted in slightly increased pressure difference values in patient 1. The results are more comparable for patient 2. Although the maximum luminal pressure difference values were almost doubled in the distal descending aorta (e.g., P6 and P7), the absolute difference between the rigid wall and FSI models was small at approximately 0.2 mmHg. Furthermore, a more compliant aortic wall of patient 1 caused higher pressure difference between two lumens, though not significant.

### Turbulence Intensity

The level of turbulence, measured in terms of turbulence intensity, was also evaluated. This is defined as

$$Tu=\;\frac{u^{\prime }}{U}$$ (8)

where U is the mean velocity and $u^{\prime }$ is the root-mean-square of the turbulent velocity fluctuations that can be expressed as:

$$u^{\prime }=\;\sqrt{\frac{1}{3}\left({u^{\prime }}_{x}^{2}+{u^{\prime }}_{y}^{2}+{u^{\prime }}_{z}^{2}\right)}=\sqrt{\frac{2}{3}k}$$ (9)

where k is the turbulence kinetic energy and therefore the turbulence intensity can be expressed as:

Tu= 23kU (10)

**FIGURE 10:**
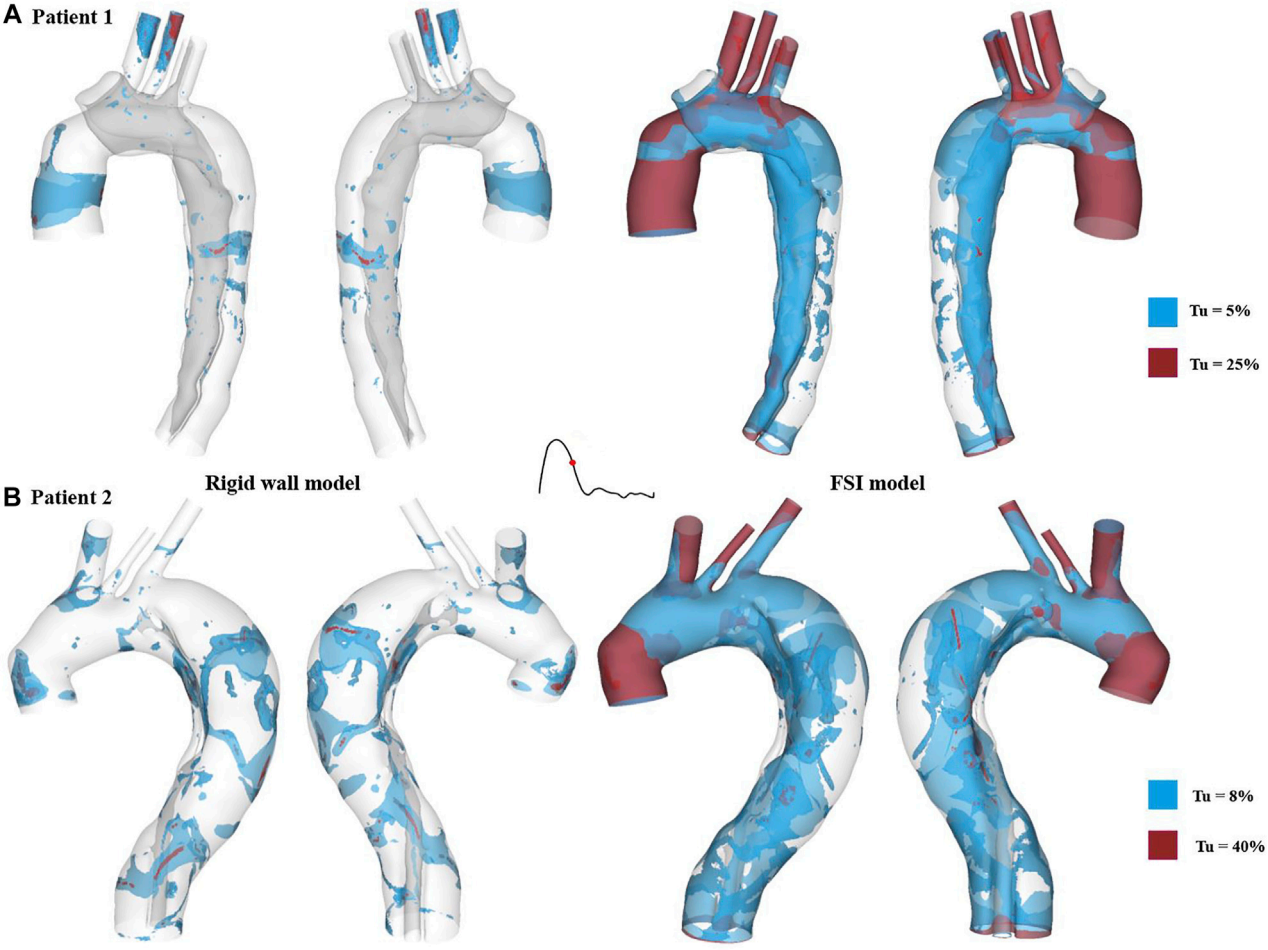
Turbulence intensity (*Tu*) iso-surfaces at mid-systolic deceleration for **(A)** patient 1 and **(B)** patient 2. *Tu* levels of approximately 5 % and 8% are shown in blue for patient 1 and patient 2, respectively, while much higher *Tu* levels are displayed in red (25% for patient 1 and 40% for patient 2).

Iso-surfaces of the turbulence intensity ( Tu ) at mid-systolic deceleration were compared between the FSI and rigid wall models, and the results for both patients are illustrated in Figure 10. It should be noted that the intensity levels presented here were based on instantaneous local velocities rather than the mean values in order to portray realistic levels. The blue and red iso-surfaces in the figure represent different values of Tu (5% and 25% for patient 1, and 8% and 40% for patient 2). In the rigid wall models, low Tu levels (shown in blue) located sporadically throughout the entire aorta, while much smaller regions presented with high Tu levels. FSI models produced greater turbulence intensity with much larger areas of iso-surfaces at higher Tu levels.

### Von Mises Stress

**FIGURE 11:**
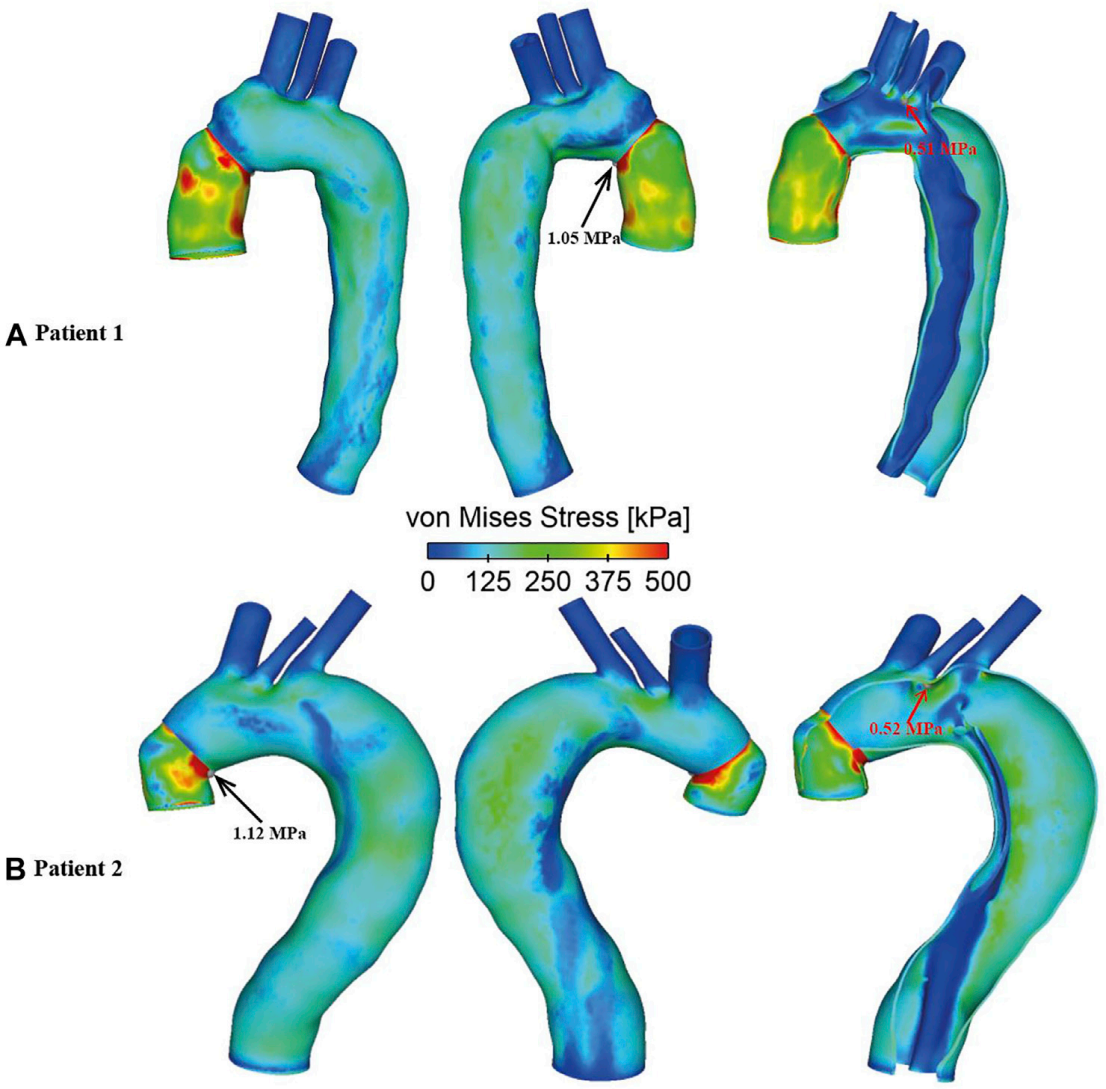
Spatial distributions of von Mises stress in FSI models for **(A)** Patient 1 and **(B)** Patient 2. Black arrows indicate the peak von Mises stress values observed at the graft-aorta interface, while red arrows indicate isolated hot spots of high von Mises stress in regions away from the graft.

Spatial distributions of von Mises stress obtained with the FSI models are shown in Figure 11, at the time point of peak systole. In both cases, peak stress values were found at the anastomosis between the graft and the aorta (as indicated by the black arrow), which could be attributed to the mismatch in material properties. Away from the graft, stress levels were much lower except at a few isolated high stress concentration spots (indicated by the red arrow) on the edge of tears and at sharp corners. The peak von Mises stress values at the graft-aorta interface increased from 1.05 to 1.15 MPa when the aortic wall Young’s modulus was reduced from 1.3 to 1.08 MPa, owing to larger difference in material properties between the graft and aortic wall.

## Discussion

Re-intervention in patients with progressive aortic dilatation after surgical repair of TAAD is typically based on aortic size and the rate of expansion. In our previous studies, a systematic examination of the flow patterns and hemodynamic factors in dissected aortas was performed, and possible links were identified between luminal pressure difference and the progression of aneurysmal dilatation (Zhu et al., 2021; Zhu et al., 2022). This study presents FSI simulations of two repaired TAAD patients, and the results from the FSI simulations are comprehensively compared with the corresponding CFD results to evaluate the assumption of rigid aortic wall. Moreover, the effect of aortic stiffness was assessed by applying various Young’s moduli to the aortic wall model of one patient.

Several studies have been published in recent years on FSI simulations of TBAD (Alimohammadi et al., 2015; Qiao et al., 2015; Chen et al., 2016; Qiao et al., 2019; Ryzhakov et al., 2019; Chong et al., 2020), in an attempt to understand the effect of wall motion on flow related parameters. Unfortunately, there were large variations in the extent of intimal flap movement incorporated in these models. Qiao et al. (2015) and Ryzhakov et al. (2019) predicted small flap displacements of up to 0.15 and 0.13 mm, respectively. In another FSI study conducted by Qiao et al. (2019), the maximum flap displacement was slightly larger than 0.6 mm, which was consistent with *in vivo* measurements of approximately 0.68 ± 0.2 mm in chronic dissections (Karmonik et al., 2012). Using an idealized model for acute dissections, Chong et al. (2020) simulated a more drastic flap motion of up to 4.6 mm. In a recent paper on residual TBAD, Bäumler et al. (2020) simulated much larger flap displacements of up to 13.4 mm, and they found that flap motion could be reduced from 13.4 to 1.4 mm by increasing the Young’s modulus from 20 to 800 kPa. The present study produced a maximum flap displacement of approximately 1 mm (Figure 2) in both patients. Decreasing aortic wall stiffness from E = 1.3 MPa to E = 1.08 MPa in patient 1 increased the maximum flap displacement from 1 mm to around 1.2 mm, which is comparable to the results reported by Bäumler et al. (2020) with the stiffest model (E_flap_ = 800 kPa).

Although the effect of wall compliance on flow patterns was negligible (Figure 3), its quantitative effect on velocity magnitudes was not trivial. In general, FSI models predicted lower blood velocities as compared to the rigid wall models, with the maximum difference among all cross-sectional planes reaching 11.5% and 11.9%, respectively, for patient 1 and 2. As mentioned above, aorta expansion during systole was responsible for reduced velocities. As shown in Figure 4A, although blood velocities were slightly increased by increasing aortic wall stiffness, the maximum difference between patient 1A (E = 1.08 MPa) and 1B (E = 2 MPa) was only 4.7%. In the rigid wall model, flow distribution to the innominate artery, left common carotid artery, and left subclavian artery was 8%, 3%, and 6%, respectively for patient 1, and 12%, 2%, and 4% for patient 2. Accounting for wall compliance did not alter the flow split among the model outlets. However, quantitative comparisons of tear flow in patient 1 revealed that blood flow entering the FL was reduced by 10.9% and 2.9%, through the primary entry tear and re-entry tear, respectively, in the FSI model. Decreasing wall stiffness from E = 1.3 MPa to E = 1.08 MPa further reduced mean flow rate at the primary entry tear and re-entry tear by 2.5% and 1.7%, respectively. Bäumler et al. (2020) reported opposite results where a more compliant flap model caused greater reduction in TL flow. However, they simulated a much wider range of flap mobility with a maximum flap displacement being almost 10 times of the displacement presented in our study. Moreover, at some cross-sectional locations, their flow split results obtained with the stiffest flap model showed better agreements with 4-D MRI data. In terms of patient 2, the amount of flow passing through all 4 re-entry tears were relatively small because of the small re-entry tear areas. Accounting for wall compliance had a notable influence on tear flow, especially at re-entry tear 4, where the mean flow rate was almost increased by three times. However, the absolute difference between the rigid wall and FSI models was only around 0.003 L/min.

Comparison of TAWSS between the rigid wall models and FSI models revealed little difference in its spatial distribution, but the magnitude of peak TAWSS predicted by the FSI model was slightly higher than (3.9%) that predicted by the rigid wall model in patient 1. In contrast to patient 1, the FSI model of patient 2 reduced the peak TAWSS magnitude by 3.6%, similar to the results reported by Alimohammadi et al. (2015) on TBAD. High WSS values have been associated with platelet activation in blood (Nobili et al., 2008), activity of which can potentially promote local thrombus formation. High WSS has also been related to degenerative lesions of the vessel wall and subsequent vessel enlargement (Ekaterinaris et al., 2006). On the other hand, Alimohammadi et al. (2015) observed that rigid wall assumption appeared to have a notable impact on the regions with low TAWSS values. In these regions, the TAWSS values were underestimated by more than 50% due to the near zero velocity values obtained by the rigid wall simulation. Therefore, regions with low TAWSS ( ≤0.2 Pa ) were identified and compared. A threshold value of 0.2 Pa was chosen since shear stresses below this value were reported to promote thrombus formation (Menichini et al., 2016). As a result of reduced velocities in the FSI models, the areas of low TAWSS regions were markedly increased in both patients when compared to the rigid models (Figure 6). Specifically, the surface areas exposed to low TAWSS were 21 and 35 cm^2^ with the rigid wall models of patients 1 and 2, respectively, increased to 38 and 144 cm^2^ with the FSI models. However, this finding is not comparable with those reported by Chong et al. (2020), who observed a reduction in the area of low TAWSS regions with the FSI model, owing to TL compression and enhanced fluid mixing in FL caused by the drastic flap motion. As shown in Figure 7, lower circumferential-averaged wall shear stress (CWSS) magnitudes were obtained with the FSI models of both patients. The maximum difference in CWSS among all selected locations along the aorta between the rigid and FSI models could reach 42.3% in the distal descending aorta (P7) of patient 2, corresponding to greater areas of low TAWSS in this region. Altering aortic stiffness had minor effects on CWSS magnitudes with the maximum difference being only 5.6% by doubling the Young’s modulus.

Blood flow was found to be highly disturbed with varying extent of flow recirculation in both true and false lumens, especially in regions surrounding the tears. Therefore, turbulence is likely to occur in the flow jet through a tear, where the peak Reynolds number can exceed 8,200 (Cheng et al., 2010). Turbulent flow within aortic aneurysms has been reported to cause extra stresses on the aneurysmal wall, increasing the rate of wall dilatation (Berguer et al., 2006; Khanafer et al., 2007). In order to capture potential turbulent flow in the post-surgery TAAD models, the SST-Tran model was adopted which allowed turbulence intensities ( Tu ) to be evaluated. As shown in Figure 10, accounting for wall compliance significantly increased Tu levels. This finding is consistent with a previous FSI study on a thoracic aortic aneurysm (Tan et al., 2009). The increase in Tu levels in the FSI results might indicate that turbulence was amplified by aortic expansion, causing sudden flow retardation.

von Mises stress is an index commonly used to identify high stress concentration spots and to assess the maximum wall stress in the aorta. The present FSI models revealed higher stress levels on the aortic wall concentrated near the tears and in highly tortuous regions, which coincided with some regions experiencing high TAWSS, indicating potential vulnerability of these regions to further increase in size. Yield stress of the dilated ascending aorta was reported to be approximately 1.2 ± 0.1 MPa in either circumferential or longitudinal directions (Vorp et al., 2003). Except a few isolated spots where extremely high stress values were found (as indicated by the red arrows in Figure 11), the aortic walls experienced wall stresses well below the threshold for rupture. Moreover, the spatial distribution of von Mises stress followed the same pattern for all simulated FSI cases with high stress concentration at the anastomosis between the graft and the native aorta, indicating a potential risk for future tear or rupture at this site.

Our previous studies have shown that higher pressure difference between TL and FL (PD_TL-FL_) may be associated with progressive aortic dilatation in repaired TAAD (Zhu et al., 2021; Zhu et al., 2022). However, one major limitation of these studies was that the CFD simulations were based on rigid wall assumptions and thereby assessing the effect of wall compliance on PD_TL-FL_ is of particular interest in this study. Most of the previous FSI studies on TBAD did not investigate the influence of wall compliance on intraluminal pressure, for example, Alimohammadi et al. (2015) and Qiao et al. (2019) analyzed wall pressure distributions and they found that TL pressure was higher than the FL pressure in the proximal region but lower in the distal region. These findings were consistent with the early numerical studies based on rigid wall assumptions (Tse et al., 2011; Cheng et al., 2015). Bäumler et al. (2020) evaluated the mean pressure difference between the TL and FL with various flap elasticities and they found that the pressure difference decreased as the flap became more compliant. However, we cannot make a direct comparison with their results because they focused on evaluating the effect of flap stiffness while keeping the aortic wall Young’s modulus constant. In our model, the aortic wall and dissection flap were assumed to have the same elastic property, so the observed differences between simulations with different Young’s moduli was most likely attributed to different wall compliance than flap mobility. Comparing the results with the rigid wall models, FSI model increased luminal pressure differences in patient 1 but decreased pressure differences in patient 2. Nevertheless, among all cross-sectional planes, the maximum difference between the FSI and rigid wall models was 0.25 and 0.2 mmHg for patient 1 and 2, respectively. This was trivial considering that unstable aortic growth was found to occur in patients with a luminal pressure difference greater than 5 mmHg (Zhu et al., 2022).

### Limitation

It is well known that the mechanical behavior of aortic wall is anisotropic and nonlinear, and the material properties of a dissected wall would vary in different components of the vessel wall, such as in TL side, FL side and intimal flap. However, for simplicity, as well as due to the lack of available data in the literature, the current study assumed the aortic wall and intimal flap to be isotropic and linear elastic with the same material property. In a recently published finite element study on TBAD, Kan et al. (2021) fitted a hyperelastic material model to their tensile testing data on dissected aortic tissues and used the hyperelastic model in finite element simulations of stent-graft deployment in TBAD. A more realistic anisotropic constitutive model has been developed by Holzapfel et al. (2000) and applied in previous finite-element studies of abdominal aortic aneurysms to analyze the wall stress distribution and rupture risks (e.g., Roy et al., 2014). However, no such study has been reported for AD since there are very limited data available regarding the material parameters for the constitutive model. A similar approach as described by Kan et al. (2021) could be adopted in the future if more relevant tensile testing data becomes available, followed by applying the anisotropic model. In addition, a constant wall thickness was assumed for the aortic wall. This was another major assumption but unavoidable because CT images do not contain sufficient information for extraction of wall thickness. Furthermore, the aortic root motion was neglected, which can influence both hemodynamic and biomechanics in different parts of the aorta. For example, Jin et al. (2003) incorporated in their CFD model both radial expansion-contraction and translational motion of the aorta at the inlet, and their results showed best agreement with the *in vivo* MR data by capturing the clockwise migration of the peak velocity zone during systole, whereas the results obtained without accounting for the root motion failed to reproduce this behaviour. On the other hand, finite element analysis of the aorta also revealed that the aortic root downward motion could significantly increase the longitudinal stress in the ascending aorta (Singh et al., 2016). Therefore, aortic root motion should be included in future simulations. Finally, although our results are comparable with relevant data in the literature, a direct validation of the numerical results with 4D-flow MRI was absent, as CT is the standard imaging modality for diagnosis of TAAD.

## Conclusion

Fully coupled two-way FSI simulations incorporating prestress were performed on two patient-specific TAAD models with surgically replaced ascending aorta. The flow patterns and TAWSS distributions were qualitatively comparable with those obtained from the rigid wall simulations, but quantitatively, FSI models reduced blood velocities and WSS magnitudes in both patients. The most notable effect of wall compliance on hemodynamic was regions with low TAWSS, which areas were increased with the FSI models by 81% and 311%, for patient 1 and 2, respectively. In addition, turbulence was significantly amplified with the FSI models, as presented by a great increase in regions with higher levels of turbulence intensity ( Tu ). Accounting for wall compliance had a much less influence on pressure difference between the true and false lumen, with the maximum difference along the aorta between the FSI and rigid wall models being only 0.25 and 0.2 mmHg for patient 1 and 2, respectively. Moreover, altering aortic wall stiffness had minor effects on all the predicted results. Although the present study offers comprehensive insights into the effect of wall compliance on hemodynamics in repaired TAAD, it would be desirable to incorporate mechanical properties representative of dissected aorta and intimal flap should these become available in the future. Finally, considering the much longer computational time for FSI simulations (approximately 8–10 times more than rigid wall CFD simulations), it may not be feasible to adopt FSI modelling in a large cohort study. Decisions on the choice of FSI or rigid wall models should be made based on the specific objectives to be accomplished. By far, rigid wall CFD simulations would be sufficient for prediction of aortic dilatation in surgically repaired TAAD based on pressure difference between the true and false lumen.

## Data Availability

The original contribution presented in the study are included in the article/Supplementary Material, further inquiries can be directed to the corresponding author.
