# Supplementary material for: Fluid-Structure Interaction Simulations of Repaired Type A Aortic Dissection: a Comprehensive Comparison With Rigid Wall Models
Source: Front Physiol. 2022 Jun 14;13:913457. doi: 10.3389/fphys.2022.913457 (PMC9237394; doi:10.3389/fphys.2022.913457)
Supplement: Supplementary file 1 [file DataSheet1.docx]

Supplementary Material

Fluid-structure interaction simulations of repaired type A aortic dissection: a comprehensive comparison with rigid wall models

Yu Zhu^1^, Saeed Mirsadraee^2,3^, Ulrich Rosendahl^2,4^, John Pepper^2,4^, Xiao Yun Xu^1*^

^1^Department of Chemical Engineering, Imperial College London, London, UK

^2^National Heart and Lung Institute, Imperial College London, London, UK

^3^Department of Radiology, Royal Brompton and Harefield Hospitals, London, UK

^4^Department of Cardiac Surgery, Royal Brompton and Harefield Hospitals, London, UK

*** Correspondence:**Professor Xiao Yun Xu
[yun.xu@imperial.ac.uk](mailto:yun.xu@imperial.ac.uk)

# **1A. Mesh Sensitivity Tests**

Mesh sensitivity tests were performed for fluid and structural domains, separately, to ensure that a mesh independent solution has been achieved.

In terms of fluid domain, transitional flow simulations with the same boundary conditions and simulation settings were carried out for different meshes in order to facilitate direct comparison of results. The simulations results were compared between different meshes in terms of maximum wall shear stress (WSS), velocity and pressure in the entire model. Moreover, three cross-sectional cut planes were created (Figure S1) in the regions around tears (regions of physiological interest and high numerical sensitivity) to compare the maximum value of spatial-mean pressure, velocity and WSS over a cardiac cycle. A mesh was considered sufficient when differences in maximum pressure, WSS and velocity between the adopted mesh and a finer mesh were less than 5%. The results of mesh sensitivity tests for the fluid domains of both patients are summarized in Table S1. It is clear that M2 containing approximately 1.55 and 1.49 million elements for post-surgical TAAD models of patient 1 and 2,respectively, should be adopted for final simulations.


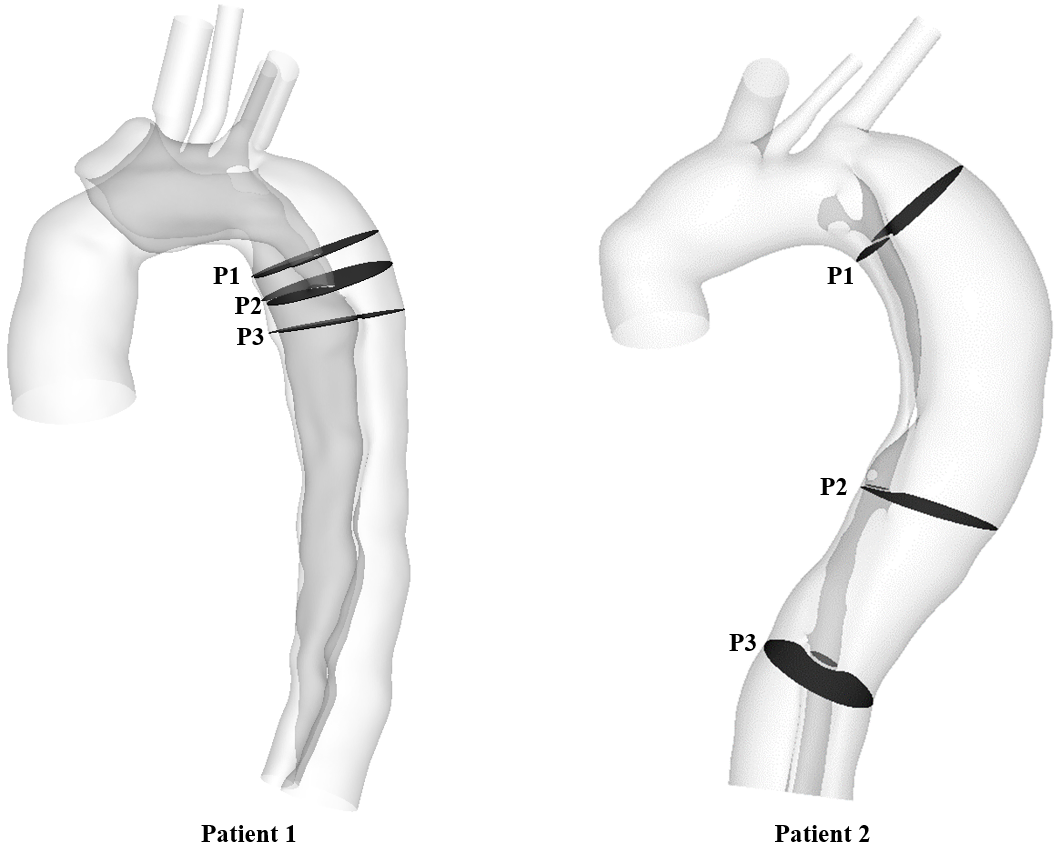


Figure S1. Three cross-sectional planes were selected in the regions near the tears, where are of physiological interest and high numerical sensitivity. The spatial-mean pressure, velocity and wall shear stress were calculated at each cut plane, after which the maximum value of each result was determined and compared between different meshes.

Table S1. Mesh sensitivity tests results showing comparison of pressure, velocity and wall shear stress (WSS) between different meshes for the fluid domain.

| **Patient 1** | | | | | |
| --- | --- | --- | --- | --- | --- |
|  | **Elements number** | **P1 (Spatial-mean)** | **P2 (Spatial-mean)** | **P3 (Spatial-mean)** | **Entire domain** |
|  |  | **Max. velocity over a cardiac cycle (m/s)** | | | |
| **M1** | 744648 | 0.34 | 0.30 | 0.20 | 1.26 |
| **M2** | 1553051 | 0.34 | 0.31 | 0.21 | 1.27 |
| **M3** | 5149928 | 0.35 | 0.31 | 0.21 | 1.27 |
| **Difference**  **(%)** | **M2/M1** | - | 3.3 | 5 | 0.8 |
|  | **M3/M2** | 2.9 | - | - | - |
|  | | **Max. pressure over a cardiac cycle (mmHg)** | | | |
| **M1** | 744648 | 101.8 | 101.9 | 102.4 | 107.4 |
| **M2** | 1553051 | 101.9 | 102.1 | 102.6 | 107.5 |
| **M3** | 5149928 | 101.5 | 101.8 | 102.3 | 107.2 |
| **Difference**  **(%)** | **M2/M1** | 0.1 | 0.2 | 0.2 | 0.09 |
|  | **M3/M2** | 0.4 | 0.3 | 0.3 | 0.3 |
|  | | **Max. WSS over a cardiac cycle (Pa)** | | | |
| **M1** | 744648 | 3.06 | 3.86 | 1.60 | 62.80 |
| **M2** | 1553051 | 3.41 | 4.03 | 1.72 | 71.62 |
| **M3** | 5149928 | 3.39 | 4.14 | 1.78 | 69.89 |
| **Difference**  **(%)** | **M2/M1** | 11.4 | 4.4 | 7.5 | 14.0 |
|  | **M3/M2** | 0.6 | 2.7 | 3.5 | 2.4 |
| **Patient 2** | | | | | |
|  | **Elements number** | **P1 (Spatial-mean)** | **P2 (Spatial-mean)** | **P3 (Spatial-mean)** | **Entire domain** |
|  |  | **Max. velocity over a cardiac cycle (m/s)** | | | |
| **M1** | 616051 | 0.19 | 0.15 | 0.19 | 0.63 |
| **M2** | 1493029 | 0.20 | 0.15 | 0.19 | 0.66 |
| **M3** | 4950759 | 0.21 | 0.15 | 0.19 | 0.67 |
| **Difference**  **(%)** | **M2/M1** | 5.3 | - | - | 4.8 |
|  | **M3/M2** | 5 | - | - | 1.5 |
|  | | **Max. pressure over a cardiac cycle (mmHg)** | | | |
| **M1** | 616051 | 93.6 | 94.2 | 94.6 | 95.0 |
| **M2** | 1493029 | 93.6 | 94.2 | 94.6 | 94.9 |
| **M3** | 4950759 | 93.5 | 94.2 | 94.6 | 94.9 |
| **Difference**  **(%)** | **M2/M1** | - | - | - | 0.01 |
|  | **M3/M2** | 0.01 | - | - | - |
|  | | **Max. WSS over a cardiac cycle (Pa)** | | | |
| **M1** | 616051 | 0.98 | 1.16 | 1.16 | 28.18 |
| **M2** | 1493029 | 1.08 | 1.19 | 1.15 | 27.41 |
| **M3** | 4950759 | 1.11 | 1.19 | 1.15 | 27.52 |
| **Difference**  **(%)** | **M2/M1** | 10.2 | 2.3 | 0.9 | 3.4 |
|  | **M3/M2** | 2.8 | - | - | 0.4 |

Regarding the structural domain, static structural analyses were performed for different meshes to compare the results of maximum displacement and von Mises stress. Solutions were considered mesh independent when the monitored parameters differed by less than 3% between two successively refined meshes. The results of mesh sensitivity tests for the fluid domains of both patients are summarized in Table S2. The results show that M2 of both patients should be chosen for final simulations.

Table S2. Mesh sensitivity tests results showing comparison of the maximum displacement and von Mises stress between different meshes for the structural domain.

| **Patient 1** | | | | |
| --- | --- | --- | --- | --- |
|  | **Elements number** | | **Max. displacement (mm)** | **Max. von Mises stress (kPa)** |
|  | **Aorta** | **Graft** |  |  |
| **M1** | 247931 | 39101 | 6.95 | 706 |
| **M2** | 846435 | 121571 | 7.31 | 761 |
| **M3** | 1988938 | 196067 | 7.51 | 782 |
| **Difference**  **(%)** | **M2/M1** | | 5.2 | 7.8 |
|  | **M3/M2** | | 2.7 | 2.8 |
| **Patient 2** | | | | |
| **M1** | 245471 | 24517 | 5.04 | 719 |
| **M2** | 843748 | 81613 | 5.28 | 725 |
| **M3** | 2115741 | 104461 | 5.44 | 732 |
| **Difference**  **(%)** | **M2/M1** | | 4.8 | 0.8 |
|  | **M3/M2** | | 3.0 | 1.0 |

# **1B. Time-step Sensitivity Tests**

Time-step sensitivity tests were also performed for fluid domains of both patients. Only two time-steps were assessed, namely 5ms and 1ms since a larger time-step would lead to unstable solutions during FSI simulations. The results shown in Table 3 indicate 5ms should be selected for final simulations.

Table S3. Time-step sensitivity tests results showing comparison of pressure, velocity and wall shear stress (WSS) between different time-steps for the fluid domain.

| **Patient 1** | | | | | |
| --- | --- | --- | --- | --- | --- |
|  | **Time-Step** | **P1 (Spatial-mean)** | **P2 (Spatial-mean)** | **P3 (Spatial-mean)** | **Entire domain** |
|  |  | **Max. velocity over a cardiac cycle (m/s)** | | | |
| **TS1** | 5ms | 0.34 | 0.31 | 0.21 | 1.26 |
| **TS2** | 1ms | 0.34 | 0.31 | 0.21 | 1.27 |
| **Difference (%)** | | - | - | - | 0.8 |
|  | | **Max. pressure over a cardiac cycle (mmHg)** | | | |
| **TS1** | 5ms | 101.8 | 102.0 | 102.4 | 107.1 |
| **TS2** | 1ms | 101.9 | 102.1 | 102.6 | 107.5 |
| **Difference (%)** | | 0.1 | 0.1 | 0.2 | 0.4 |
|  | | **Max. WSS over a cardiac cycle (Pa)** | | | |
| **TS1** | 5ms | 3.37 | 4.05 | 1.71 | 72.37 |
| **TS2** | 1ms | 3.41 | 4.03 | 1.72 | 71.62 |
| **Difference (%)** | | 1.2 | 0.5 | 0.6 | 1.0 |
| **Patient 2** | | | | | |
|  | **Elements number** | **P1 (Spatial-mean)** | **P2 (Spatial-mean)** | **P3 (Spatial-mean)** | **Entire domain** |
|  |  | **Max. velocity over a cardiac cycle (m/s)** | | | |
| **TS1** | 5ms | 0.20 | 0.15 | 0.19 | 0.66 |
| **TS2** | 1ms | 0.20 | 0.15 | 0.19 | 0.66 |
| **Difference (%)** | | - | - | - | - |
|  | | **Max. pressure over a cardiac cycle (mmHg)** | | | |
| **TS1** | 5ms | 93.5 | 94.2 | 94.6 | 94.9 |
| **TS2** | 1ms | 93.6 | 94.2 | 94.6 | 94.9 |
| **Difference (%)** | | 0.1 | - | - | - |
|  |  | **Max. WSS over a cardiac cycle (Pa)** | | | |
| **TS1** | 5ms | 1.08 | 1.21 | 1.18 | 27.50 |
| **TS2** | 1ms | 1.08 | 1.19 | 1.15 | 27.41 |
| **Difference (%)** | | - | 1.7 | 2.5 | 0.3 |
